# Mitochondrial- and Endoplasmic Reticulum-Associated Oxidative Stress in Alzheimer's Disease: From Pathogenesis to Biomarkers

**DOI:** 10.1155/2012/735206

**Published:** 2012-06-04

**Authors:** E. Ferreiro, I. Baldeiras, I. L. Ferreira, R. O. Costa, A. C. Rego, C. F. Pereira, C. R. Oliveira

**Affiliations:** ^1^Center for Neuroscience and Cell Biology (CNC), University of Coimbra, Largo Marquês de Pombal 3004-517, Coimbra, Portugal; ^2^Faculty of Medicine, University of Coimbra, Rua Larga 3004-504, Coimbra, Portugal; ^3^University Coimbra Hospital, 3000-075, Coimbra, Portugal

## Abstract

Alzheimer's disease (AD) is the most common cause of dementia in the elderly, affecting several million of people worldwide. Pathological changes in the AD brain include the presence of amyloid plaques, neurofibrillary tangles, loss of neurons and synapses, and oxidative damage. These changes strongly associate with mitochondrial dysfunction and stress of the endoplasmic reticulum (ER). Mitochondrial dysfunction is intimately linked to the production of reactive oxygen species (ROS) and mitochondrial-driven apoptosis, which appear to be aggravated in the brain of AD patients. Concomitantly, mitochondria are closely associated with ER, and the deleterious crosstalk between both organelles has been shown to be involved in neuronal degeneration in AD. Stimuli that enhance expression of normal and/or folding-defective proteins activate an adaptive unfolded protein response (UPR) that, if unresolved, can cause apoptotic cell death. ER stress also induces the generation of ROS that, together with mitochondrial ROS and decreased activity of several antioxidant defenses, promotes chronic oxidative stress. In this paper we discuss the critical role of mitochondrial and ER dysfunction in oxidative injury in AD cellular and animal models, as well as in biological fluids from AD patients. Progress in developing peripheral and cerebrospinal fluid biomarkers related to oxidative stress will also be summarized.

## 1. General Introduction

Alzheimer's disease (AD) is the most common form of dementia with a progressive course. AD pathology evidences neuronal damage in specific vulnerable brain regions and circuits involved in memory and language, namely, the hippocampus and cerebral cortex, which appears to be preceded by synaptic and neuronal dysfunction. From a pathology perspective, the presence of extracellular plaques, mainly composed of amyloid beta peptide (A*β*), a 39- to 42-aminoacid residue peptide, derived from the processing of amyloid precursor protein (APP), and intraneuronal neurofibrillary tangles, consisting of tau protein aggregates, constitute, important hallmarks of the disease and serve, as a dividing line between AD and other dementias [1–4]. Demented individuals who do not have plaques and tangles does not qualify for a diagnosis of AD, but the simple presence of plaques and tangles do not distinguish demented from nondemented individuals since brains of aged nondemented individuals frequently contain plaques and tangles [3].

Although the etiology of AD is largely unknown, it has been hypothesized that multiple factors, including genetic components, oxidative stress, intracellular and/or extracellular accumulation of A*β*, excitotoxicity, inflammation, mitochondrial dysfunction, alteration of cytoskeleton and synapse components and neuronal loss, may play important roles in the onset of the disease [5]. One hypothesis that may account for the heterogeneous nature of AD and the fact that aging is the most obvious risk factor is the increased generation of reactive oxygen species (ROS); indeed, neurons are extremely sensitive to attack by destructive free radicals [6].

## 2. Evidence of Oxidative Stress in AD Brain

The “oxidative stress theory” of aging holds that a progressive and irreversible accumulation of oxidative damage caused by ROS impacts on critical aspects of the senescence process, contributing to impaired physiological function, increasing incidence of disease, along with a reduction in life span [7]. Although low and intermediary levels of ROS are physiologically important, high ROS concentrations above the clearance capacity of the cell cause oxidative stress, mitochondrial dysfunction, cellular damage, and, in numerous cases, cell death [8], thus pointing oxidative stress as a potential unifying mechanism contributing to age-related pathologies [7] and, in particular, to AD [9, 10].

Lipid peroxidation is one of the major outcomes of free-radical-mediated injury leading to the generation of a variety of relatively stable end products. The ones that have been most extensively studied, both in brain and biological fluids, such as cerebrospinal fluid (CSF), plasma, urine of AD and mild cognitive impairment (MCI) patients, are malondialdehyde (MDA), trans-4-hydroxy-2-nonenal (HNE), and F_2_-isoprostanes (F_2_-IsoPs). Indeed, several studies have demonstrated significantly increased levels of MDA and thiobarbituric acid reactive substances (TBARS) in AD [11–13] and MCI brains [14], particularly in regions where neurofibrillary tangles and senile plaques typically accumulate. HNE, one of the most toxic products of lipid peroxidation, is, like MDA, diffusible and highly reactive with other biomolecules being able to covalently modify proteins, thus affecting their function. Increased levels of free HNE and HNE-protein adducts have been described in the brains of MCI and AD patients compared to controls [15–19]. In addition, increased levels of F_2_-IsoPs have been documented in different brain regions of AD in comparison to cognitively normal individuals [20–22]. This increase of F_2_-IsoPs was demonstrated to be specific of AD-type dementia and did not occur in cases of frontotemporal dementia [21]. F_2_-IsoPs have also been investigated in brain of MCI subjects. Increased levels of these lipid peroxidation products were documented in different brain regions of MCI subjects compared to controls [23]; however these data were not confirmed by other authors [24].

Within proteins, all amino acids can be attacked by ROS, but sulphur-containing and aromatic amino acids are the most susceptible. The oxidation of amino acids mainly leads to the formation of carbonyl groups, while peroxynitrite can nitrate tyrosine groups and form the stable compound 3-nitrotyrosine (3-NT). Increased levels of protein carbonyls have been detected in the superior and middle temporal gyri of patients with early-stage AD and MCI and also in the hippocampus and parietal lobe of AD patients compared to controls [14, 25, 26], but unchanged in the cerebellum, which is consistent with the regional pattern of histological changes in AD. On the other hand, increased 3-NT immunoreactivity has been also detected in regions of the cerebral cortex affected by neurodegeneration in AD patients [27], with a distribution similar to protein carbonyls. Moreover, high levels of protein nitration were found in inferior parietal lobes and hippocampi of MCI patients [28]. Protein oxidation in AD does not seem to be a random process but rather involves specifically more susceptible proteins that have been identified through redox proteomic studies [29]. Many of the proteins that have been identified so far, as oxidatively modified in the brain of AD patients and MCI subjects, are either mitochondrial proteins or proteins that are known to interact with mitochondria; these include glyceraldehyde 3-phosphate dehydrogenase (GAPDH), voltage-dependent anion channel (VDAC), lactate dehydrogenase (LDH), malate dehydrogenase (MDH), adenosine triphosphate (ATP) synthase-alpha chain, beta-actin and/or aconitase [18, 30–32].

ROS, and particularly the hydroxyl radical, can react with all components of the DNA molecule, causing different kinds of damage. DNA injury has been investigated in AD and MCI subjects mainly through the analysis of DNA strand breaks and the presence of specific oxidized DNA bases and adducts, of which 8-hydroxy-2′-deoxyguanosine (8-OHdG) is the most commonly investigated. Several postmortem studies have reported significant DNA fragmentation in the brain of AD subjects compared to nondemented controls, especially in areas that are more prone to neurodegeneration [33–36]. A buildup of 8-OHdG was detected in brain tissue from AD subjects, that was most prominent in mitochondrial DNA (mtDNA) of the parietal cortex [37]. These results were confirmed by another report showing that the presence of oxidized nucleosides was inversely related to the neurofibrillary tangle content [38], further suggesting that DNA oxidation could precede lesion formation. This hypothesis was further corroborated by a study by Wang and coauthors [39] who observed higher indices of oxidation in mtDNA from neocortical regions of MCI subjects compared to controls, but similar to the ones observed in AD patients, suggesting that DNA oxidation was indeed an early event in the pathogenesis of the disease. RNA is more vulnerable to oxidation than DNA and can be easily attacked by the hydroxyl radical. Several studies evaluated the levels of 8-hydroxyguanosine (8-OHG) as a marker of oxidative damage to RNA. Immunohistochemical analysis of neurons in particularly vulnerable brain areas of AD patients showed a marked accumulation of 8-OHG, that was negatively correlated with the duration of the disease and the extent of A*β* deposition [40]. These findings have been further extended by Shan and collaborators that showed a large increase in the extent of messenger RNA (mRNA) oxidation in the frontal cortex, but not in the cerebellum of AD patients [41, 42]. It was also demonstrated that increased levels of 8-OHG in the parahippocampal gyrus were already present in MCI subjects, compared to controls, but similar to the levels found in AD patients [43], suggesting that RNA oxidative damage is an early event in AD pathology.

Very recently, multiple biochemical markers of oxidative stress and antioxidant defenses were analyzed in frontal cortex postmitochondrial supernatant, mitochondrial, and synaptic fractions from age-matched noncognitively impaired, mild cognitive impairment (MCI), mild AD, and AD subjects [44]. In this study, a strong correlation was observed between levels of synaptic lipid peroxidation, protein oxidation and nitration, and the subjects' global cognitive status. Changes in levels of the antioxidants glutathione (GSH), superoxide dismutase (SOD), and catalase (CAT) also strongly correlated with the minimental status examination (MMSE) score [45]. Previous studies found both increased [11] and reduced activity of antioxidant enzymes in AD [12] and MCI brain [46].

In studies assessing oxidative damage in brain, the possibility of artifacts due to postmortem delay cannot be completely ruled out. However, in most of the referred studies postmortem interval was conveniently short (1–5 hours), matched between patients and control samples and therefore should not have a significant effect on the discussed parameters. In fact, a few studies [33, 47] have examined the influence of postmortem delay in oxidative damage measures, and similar levels have been found in rapid (<1 h) and conventional autopsy tissue (up to 8 hours). Overall, these findings support the idea that the unbalance between ROS generation and detoxification by antioxidants is an early event that plays an important role in the progression of the disease.

## 3. ROS Generation and Mitochondrial Dysfunction

In cells, multiple pathways and enzymes can generate ROS. These include, as an example, complexes I and III of the mitochondrial respiratory chain in the mitochondrion, nicotinamide adenine dinucleotide phosphate (NADPH) oxidase (NOX), xanthine oxidase, or nitric oxide synthase (NOS) [8]. Mitochondria produce ROS and reactive nitrogen species (RNS) during the normal aerobic activity. This accounts for the generation of superoxide (O_2_^•^^−^), mainly produced at complex I and complex III of the electron transport chain, and nitric oxide (^•^NO). ^•^NO controls mitochondrial respiration and both cytotoxic, as well as cytoprotective effects have been described to be due to this RNS. Depression of ATP synthesis through oxidative phosphorylation by ^•^NO has been mainly attributed to the inhibition of mitochondrial complex IV. In fact, ^•^NO-induced inhibition of complex IV is completely and quickly reverted upon its removal, suggesting that the inhibition of mitochondrial complex IV by ^•^NO can be better described as a functional control of cell respiration [48]. Importantly, if these two molecules (O_2_^•^^−^ and ^•^NO) encounter each other, they undergo a fast spontaneous reaction leading to production of peroxynitrite (ONOO^−^). For this purpose, classical antioxidant pathways, such as superoxide dismutase (SOD2 in the matrix and also SOD1 at the intermembrane space) and the glutathione cycle, play a relevant role in detoxifying increased mitochondrial ROS levels. Although it is unclear whether the decline in antioxidants precedes the increase in oxidants during AD progression, their levels are certainly not capable of neutralizing enhanced ROS generation [44]. Thus, mitochondria require efficient expression of antioxidant enzymes. In this perspective, oxidative stress is also seen as an imbalance that has its origins in genes and in the way in which gene expression is regulated. At the center of this new focus is a transcription factor named nuclear factor (erythroid-derived 2)-like 2, or Nrf2 (described further in this paper), the “master regulator” of the antioxidant response, modulating the expression of hundreds of genes, including the familiar antioxidant enzymes [49].

Evidence from AD postmortem brain, as well as cellular and animal AD models, shows that A*β* triggers mitochondrial dysfunction by interaction with different mitochondrial targets, including the outer mitochondrial membrane OMM, intermembrane space, inner mitochondrial membrane IMM, and the matrix. The consequent impairment of oxidative phosphorylation, ROS production, mitochondrial dynamics, and the interaction with mitochondrial proteins [50] may be related to a toxic effect caused by intracellular A*β*. Indeed, A*β* has been described to accumulate intracellularly, a process linked to early stages in the neuropathological phenotype of AD [51]. Within the cells, aggregated A*β*1-42 may appear as dense packed granules [52]. Moreover, intracellular A*β* is present in mitochondria from brains of AD transgenic mice and AD patients. A*β* progressively accumulates in mitochondria and is associated with decreased activity of complexes III and IV and a reduction in the rate of oxygen consumption [53]. Importantly, A*β* can be transported into mitochondria via the translocase of the outer membrane (TOM) machinery in a process independent of the mitochondrial membrane potential [54].

Concordantly, many studies have shown mitochondrial abnormalities in AD, as expressed both by energy deficits and the potentially toxic production of free radicals [6]. Imaging and biochemical studies in brain and peripheral samples obtained from AD patients revealed alterations in both extramitochondrial and mitochondrial metabolic pathways. Accordingly, reduced cerebral glucose transport and pyruvate levels through glycolysis were observed in the temporal cortex of AD subjects. Moreover, deregulation of tricarboxylic acid cycle and oxidative phosphorylation system coupled to altered mitochondrial dynamics were also found [55, 56], along with the well-defined deficit in mitochondrial complex IV [57]. Thus, mitochondria are susceptible organelles in AD, largely contributing for disease-related ROS generation and AD pathogenesis.

Both mitochondrial ROS production and Ca^2+^ handling (which is necessary for the activity of mitochondrial dehydrogenases) are considered the centre of important biological processes, and their deregulation has been implicated in a number of human pathologies, including neurodegenerative diseases like AD. Due to localized high Ca^2+^ concentration in microdomains close to mitochondria, Ca^2+^ is rapidly accumulated within mitochondria (e.g., [58]) influencing energy function by activating mitochondrial matrix dehydrogenases to produce more NADH, donating more electrons through complex I, and thus driving the synthesis of ATP. Thus, the role of mitochondria as reservoirs of Ca^2+^ and apoptotic proteins and producers of ROS is pathologically linked to neurotoxicity in both AD and aging brain. However, most investigators agree that mitochondria from AD subjects differ from those of age-matched, nondemented subjects [3, 59–61]. The role of mitochondrial ROS as inducers of Ca^2+^ deregulation is well established, and a major cause of ROS production has been linked to Ca^2+^ deregulation, along with reduced mitochondrial ATP levels. Thus, oxidative stress and Ca^2+^ regulation are intricately linked and can cooperatively contribute to AD pathogenesis [60, 61].

Apart from producing ROS and RNS, mitochondria are susceptible targets for oxidant molecules. These can attack mitochondrial lipids, proteins, and DNA. In fact, the lack of histones in mtDNA renders them vulnerable organelles to oxidative stress [7, 8]. Mitochondrial-targeted ROS scavengers, without interfering with physiological ROS signaling, therefore represent a promising novel therapeutic approach to the treatment of neurodegenerative diseases like AD [8, 60]. In recent studies the mitochondrial antioxidant MitoQ (mitoquinone mesylate: [10-(4,5-dimethoxy-2-methyl-3,6-dioxo-1,4-cycloheexadienl-yl) decyl triphenylphosphonium methanesulfonate]) prevented increased production of ROS and the loss of mitochondrial membrane potential in cortical neurons subjected to A*β* and further prevented cognitive decline, synaptic loss, caspases activation, and oxidative stress in female of 3xTg-AD mice [62].

To better access mitochondrial dynamics and how A*β* affects the function of this organelle, researchers mainly use *in vitro* strategies. In pyramidal neurons from the hippocampus of AD patients, the levels of intracellular A*β*1-40 and −42 were found to be 3 and 10 *μ*M, respectively, higher than those found in control individuals [63], which are in the range of the concentrations used in numerous *in vitro* studies. In fact, by using isolated rat brain mitochondria treated with A*β*, both mitochondrial transmembrane potential and the mitochondrial capacity to accumulate Ca^2+^ were shown to be decreased and to cause a complete uncoupling of respiration [64]. Moreover, mitochondrial accumulation of A*β* reduced oxygen consumption and mitochondrial electron transport chain activity [65, 66]. The progressive accumulation of A*β* within this organelle was shown to be linked to mitochondrial abnormalities, like mtDNA defects and altered mitochondrial gene expression, along with changes in mitochondrial dynamics [67], axonal transport, and also synaptic degeneration [50, 60].

Deregulated Ca^2+^ levels are also detrimental to mitochondrial function, and therefore impaired Ca^2+^ homeostasis may play a role in ROS generation, A*β* aggregation, and damage to mitochondria in AD [68]. A*β* can further promote intracellular Ca^2+^ increase in a deleterious positive feedback loop [69], suggesting that A*β* accumulation can deregulate Ca^2+^ levels and vice versa. In fact, L-, P- and N-type Ca^2+^ channels activity can be modulated by A*β*, an effect apparently mediated primarily by A*β*-induced ROS production [68]. A*β* was also shown to promote excessive release of Ca^2+^ from endoplasmic reticulum (ER), which may underlie mitochondrial Ca^2+^ dyshomeostasis and ROS generation, thereby disturbing organelle functioning and, ultimately, damaging neurons [55], as described above.

The mild or gradual energy disturbance, described above, may influence ROS generation (namely, through disruption of the mitochondrial respiratory chain) and cause the oxidative damage of different molecules and the formation of the high conductance mitochondrial cyclophilin D-associated permeability transition pore (PTP) [70]. This is followed by the release of proapoptotic factors, particularly cytochrome c and apoptosis-inducing factor (AIF) and the activation of caspases in charge of the “execution” phase of the apoptotic cascade [71]. In this perspective, apoptosis through the intrinsic pathway has been largely described to play an essential role in AD pathogenesis [72]. In response to apoptotic signals, loss of mitochondrial membrane potential associates with mitochondrial membrane permeabilization to evoke cytochrome c release and the activation of the initiator caspase-9. Nevertheless, evidence of apoptosis has been largely controversial in AD. Although many reports support the occurrence of mitochondrial-linked apoptosis, as observed following exposure to A*β*, other researchers have not seen an increase in apoptosis. Previous reports described that the hippocampus of AD brains displayed DNA fragmentation, but only few cells showed morphological characteristics of apoptosis [73]. This has been opposed by studies in cell and animal models of AD overexpressing the antiapoptotic protein Bcl-2. In this regard, we previously showed that Bcl-2 is neuroprotective against apoptotic cell death caused by A*β*(25–35) [74]. Additionally, overexpression of Bcl-2 in 3xTg-AD mice improved place recognition memory, reduced caspase activation, and attenuated APP processing, leading to decreased formation of extracellular plaques and neurofibrillary tangles [75].

### 3.1. Oxidative Stress and Synaptic Loss: The Relevance of Synaptic Mitochondria

Synapses are sites of high energy demand and extensive Ca^2+^ fluctuations since synaptic transmission requires high levels of ATP and constant regulation of intracellular Ca^2+^ concentration, rendering synaptic mitochondria vital for maintenance of synaptic function and transmission [59].

Recent studies in postmortem frontal cortex obtained from MCI individuals or mild/moderate and late-stage AD patients demonstrated a significant disease-dependent increase in oxidative markers mainly localized to the synapses. Interestingly, the levels of oxidative markers significantly correlate with MMSE suggesting an involvement of oxidative stress in AD-related synaptic loss [44]. A recent study also demonstrated mitochondrial morphologic alterations in neurons obtained from different brain areas of postmortem human AD brains concomitantly, with loss of dendritic branches and depletion of dendritic spines [76]. In AD, synaptic dysfunction and the loss of synapses are in fact early pathological features, probably due to defects in synaptic mitochondria, which lead to alterations in cognitive function [44], and, interestingly, this seems to be related to ROS production and altered Ca^2+^ dynamics at the synapse [61]. In mouse hippocampal neurons, A*β* was demonstrated to impair mitochondrial movements, reduce mitochondrial length, and cause synaptic degeneration [77]. Compared with nonsynaptic mitochondria, synaptic mitochondria showed a greater degree of age-dependent accumulation of A*β* and mitochondrial alterations. The fact that synaptic mitochondria, especially A*β*-rich synaptic mitochondria, are more susceptible to A*β*-induced damage highlights the central importance of synaptic mitochondrial dysfunction to the development of synaptic degeneration in AD [59]. Indeed, synaptic mitochondria are more sensitive to ROS than nonsynaptic mitochondria [78].

In AD, synapses are the primary sites of Ca^2+^ deregulation due to overactivation of glutamate receptors. These receptors are concentrated on postsynaptic spines of neuronal dendrites where they are subjected to particularly high levels of Ca^2+^ influx, oxidative stress, and ATP demand. Therefore, they are likely sites at which neurodegenerative processes are initiated in aging and early AD, thus playing an important role in decreased synaptic function. In addition, the apoptotic process has been shown to be activated locally in synaptic compartments after exposure to A*β* in vulnerable AD neuronal populations [79].

With this in mind, in the next section we discuss the role of *N*-methyl-*D*-aspartate receptors (NMDARs), a subtype of glutamate receptors, in mitochondrial Ca^2+^ regulation and ROS formation in AD-associated neurodegeneration.

### 3.2. Role of NMDA Receptors in AD

**Figure 1:**
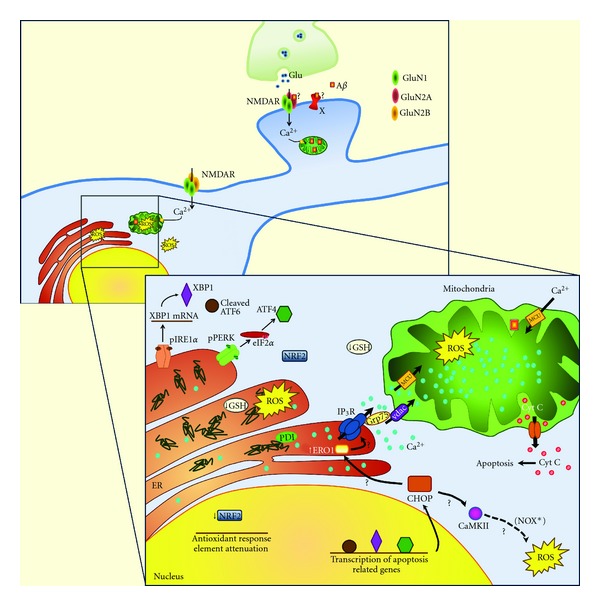
Sources of reactive oxygen species in Alzheimer's disease. Extracellular accumulation of A*β* may direct or indirectly alter NMDARs-mediated glutamatergic neurotransmission with concomitant cytosolic Ca^2+^ increase and impaired synaptic activity. Excitotoxic increase in glutamatergic neurotransmission may activate extrasynaptic NMDARs leading to a massive increase in the intracelular Ca^2+^, which is rapidly taken up by mitochondria and ER. Mitochondria Ca^2+^ overload promotes the generation of ROS. Additionally, the ER may also promote ROS production. Decreased PDI activity may lead to polyubiquitinated proteins accumulation, which may thus induce the UPR, mediated by IRE1*α*, PERK, and ATF6 pathways. In order to cope with the need to balance disulfide bond formation, the activity of ERO1*α* is increased leading to the production of ROS that are able to directly attack and affect IP_3_R function. Since the ER and the mitochondria are in close proximity, the Ca^2+^ released from the ER, through the IP_3_R, can then enter directly into mitochondria, through the VDAC or the MCU, leading to the increase in mitochondrial Ca^2+^ content, inducing mitochondrial ROS production. As a result of prolonged ER stress, CHOP may induce ERO1*α* upregulation or activate the enzyme CaMKII, which can further activate NOX, localized at the plasma membrane, enhancing cytosolic ROS production. As a consequence, protective antioxidant defenses such as GSH are depleted. In addition, Nrf2, which normally translocates to the nucleus where it activates the antioxidant response element, may be retained in the cytosol.

Ionotropic glutamate receptors mediate most excitatory neuronal transmission in the brain and play essential roles in the regulation of synaptic activity. In fact, Ca^2+^ influx through NMDARs induced by synaptic activity is required for many types of synaptic plasticity and underlies some forms of learning and memory. Very recently, the selective roles for GluN2A and GluN2B subunits of the NMDARs in long-term potentiation (LTP) and long-term depression (LTD), respectively, were reported [80]. However, excessive Ca^2+^ influx due to overactivation of NMDARs may result in excitotoxic cell death in many neurological disorders, including AD [81] (Figure 1).

Depending on their specific response to different pharmacological agents, ionotropic glutamate receptors can be subdivided into NMDARs, *α*-amino-3-hydroxy-5-methyl-4-isoxazolepropionic acid (AMPA) and kainate receptors [81, 82]. A*β* oligomers were shown to induce inward currents, intracellular Ca^2+^ increase, mitochondrial Ca^2+^ overload, oxidative stress, mitochondrial membrane depolarization, and apoptotic cell death through a mechanism requiring NMDAR and AMPAR activation in both rat cortical neurons and hippocampal organotypic slices [69].

Functional NMDARs are heterotetramers composed of two glycine-binding GluN1 subunits assembling with two glutamate-binding GluN2 (GluN2A–GluN2D) subunits or, alternatively, GluN3 (GluN3A and/or GluN3B) subunits which can replace GluN2 [83]. The most widely expressed NMDARs contain the obligatory subunit GluN1 plus either GluN2B or GluN2A or a mixture of the two. GluN2B and GluN2D subunits are expressed at high levels in early developmental stages (prenatally), whereas GluN2A and GluN2C expression is first detected near birth [84]. NMDARs exhibit high Ca^2+^ permeability and voltage-dependent channel block by extracellular Mg^2+^ [81], properties of both physiological and pathological importance. Channel blockade by Mg^2+^ reduces Ca^2+^ influx at membrane voltages near rest but is relieved during neuronal excitation [81].

Recent studies have reported activation of the ROS-producing NOX after NMDAR stimulation in response to intrastriatal administration of glutamate in mice. In contrast, mice lacking NOX2 were less vulnerable to excitotoxicity, presented reduced levels of ROS production and protein nitrosylation, decreased microglial reactivity and calpain activation, suggesting that NOX is stimulated by Ca^2+^ entry through ionotropic glutamate receptors [85]. Recent results also demonstrate that not only glutamate excitotoxicity and/or oxidative stress alter mitochondrial fission/fusion, but that an imbalance in mitochondrial fission/fusion in turn leads to NMDAR upregulation and oxidative stress [86], suggesting a new vicious cycle involved in neurodegeneration that includes glutamate excitotoxicity, oxidative stress, and mitochondrial dynamics.

Although NMDARs activation is essential for memory formation, therapeutic actions of memantine, an uncompetitive open channel blocker of NMDARs, include slowing of neuronal loss due to NMDARs excitotoxicity, thus correcting for an excitation-inhibition imbalance. Indeed, memantine is widely prescribed as a memory-preserving drug for moderate- to late-stage AD patients [87], suggesting that the therapeutic effect of memantine derives predominantly from NMDARs inhibition. However, it appears paradoxical that inhibition of NMDARs slows memory loss associated with AD, considering that NMDARs activation is essential for memory formation.

A*β* oligomers were previously reported to coimmunoprecipitate with extracellular domains of the GluN1 subunit, suggesting a direct interaction of A*β* with NMDARs [88]. Using transfected HEK293 cells, it has previously been shown that A*β* mediates necrotic cell death through changes in Ca^2+^ homeostasis in HEK293 cells selectively expressing GluN1/GluN2A subunits, but not GluN1/GluN2B subunits [84]. However, in rat primary cortical cultures it was recently demonstrated that A*β*1-42 preparation containing both oligomers (in higher percentage) and monomers directly interacts with cell function by disturbing intracellular Ca^2+^ homeostasis through activation of GluN2B-containing NMDARs [89]. Moreover, the same preparation of A*β*1-42 induced microtubule disassembly, reduced neurite length and DNA fragmentation in mature hippocampal cells, which were largely prevented by the selective NMDAR antagonists MK-801 (noncompetitive antagonist), memantine and ifenprodil (GluN2B subunit antagonist), suggesting a role for extrasynaptic GluN2B-containing NMDARs in A*β* toxicity, as recently shown by Mota and colleagues (in press).

Application of A*β* monomers and low-n oligomers (dimers and trimers) secreted from Chinese hamster ovary cells that stably overexpress human APP bearing the Val717Phe familial AD mutation was shown to mimic a state of partial NMDAR blockade, reducing NMDAR activity and NMDAR-dependent Ca^2+^ influx [90]. Accordingly, neurons from a genetic mouse model of AD were found to express reduced amounts of surface GluN1 subunit [91], and A*β*1-42 was also found to reduce surface expression of the GluN1 subunit, in both cortical and hippocampal neurons [91, 92]. On the other hand, GluN2A- and GluN2B-NMDARs appear to have opposite roles in regulating intracellular Ca^2+^ in the presence of A*β*1-42 in rat cortical cultures [89]. These findings support the concept that dysregulation of intracellular Ca^2+^ homeostasis is induced by a possible interaction of A*β* with NMDARs, particularly of the GluN2B subtype. In addition, it was also demonstrated that in the AD brain and human cortical neurons, excitatory synapses containing the GluN2B subunit of the NMDAR appear to be the main sites of oligomer accumulation. In this study, A*β* oligomers colocalized with synaptic markers, and this effect was counteracted by ifenprodil and memantine, blocking the ion channel formed by the NMDAR [93].

There is a growing body of evidence that NMDAR activity has the potential to promote survival or death in neurons of the central nervous system [94], which may be related to differences in synaptic versus extrasynaptic NMDAR signaling. It was recently demonstrated that extrasynaptic, but not synaptic, NMDARs activity stimulates neuronal amyloidogenic *β*-secretase-mediated APP processing and increases A*β* production in primary cultures of cortical neurons [95]. Interestingly, in this study, memantine inhibited extrasynaptic NMDAR-induced APP protein expression as well as neuronal A*β* release in a dose-dependent manner. In fact, the differences between synaptic and extrasynaptic pools could be due to the way they are activated: brief saturating activation in the case of synaptic NMDARs, compared with chronic, low-level activation of extrasynaptic NMDARs by bath application of glutamate. Differences in the properties of intracellular Ca^2+^ transients evoked by these different stimuli may differentially affect signaling, even if the overall Ca^2+^ load is similar [96, 97].

Ca^2+^ influx through NMDARs activation also seems to have opposite consequences on neuronal fate, according to their cellular localization [98, 99]. Stimulation of synaptic NMDARs induces prosurvival events through the activation of cAMP response element-binding protein (CREB) [100] and the extracellular signal-regulated kinase (ERK) cascade [101]. Conversely, Ca^2+^ influx through extrasynaptic NMDARs overrides these functions coupling to a dominant CREB shut-off pathway causing CREB dephosphorylation, which is less well tolerated, triggering decreased mitochondrial membrane potential and cell death [99]. Thus, a distinct NMDARs activation signaling pathway was postulated, depending on their localization. Synaptic stimuli evoke Ca^2+^entry through both GluN2A- and GluN2B-containing NMDARs and, in contrast to excitotoxic activation of extrasynaptic NMDARs, produce only low-amplitude cytoplasmic Ca^2+^ spikes and modest nondamaging mitochondrial Ca^2+^ accumulation [102]. However, NMDAR signaling can also be due to differences in the composition of the NMDARs as opposed to the location of the receptors. Thus, it has been suggested that excitotoxicity is triggered by the selective activation of NMDARs containing the GluN2B subunit [103, 104] irrespective of its location (synaptic or extrasynaptic), as GluN2A-containing NMDARs promote survival [104]. Accordingly, Ca^2+^ entering through GluN2A or GluN2B subunits-containing NMDARs was shown to have antiapoptotic activity or mitochondrial dysfunction and cell death, respectively [100].

## 4. ER and Oxidative Stress in AD

### 4.1. ER Stress and ER-Mitochondria Crosstalk in AD

#### 4.1.1. ER Stress in AD

The ER is a multifunctional organelle that plays a central role in many essential cellular activities, such as folding, assembly and quality control of secretory and membrane proteins, disulfide bond formation, glycosylation, lipid biosynthesis, Ca^2+^ storage and signaling. Under stress conditions, such as perturbed Ca^2+^ homeostasis or redox status, elevated secretory protein synthesis rates, altered glycosylation levels, and hypercholesterolemia, unfolded or misfolded proteins accumulate in the ER lumen leading to ER stress [105]. To relieve stress and reestablish homeostasis, the ER activates intracellular signal transduction pathways, collectively termed the unfolded protein response (UPR), which reduces the influx of newly synthesized proteins into the ER through induction of general translational arrest and induces the transcriptional upregulation of genes that enhance the ER protein-folding capacity and quality control. During UPR, the ER also employs proteasomal (ER-associated degradation, ERAD) and autophagic pathways to degrade mis- or unfolded proteins [106]. Three specialized ER stress-sensing proteins involved in the canonical mammalian UPR pathway have been identified: protein kinase R-like endoplasmic reticulum kinase (PERK), inositol-requiring enzyme 1 *α* (IRE1*α*) and activating transcription factor 6 (ATF6). Upon ER stress, the ER chaperone glucose-regulated protein 78 (Grp78) dissociates from these ER transmembrane sensors and promotes their activation, inducing phosphorylation and oligomerization of IRE1, and PERK, and translocation of ATF6 to the Golgi where it is cleaved by Site 1 and Site 2 proteases (S1P and S2P). Active IRE1*α* processes the mRNA encoding X-box binding protein 1 (XBP1), a transcription factor that upregulates genes encoding mediators of protein folding, ERAD, organelle biogenesis, and protein quality control. PERK activation reduces protein load in the ER by decreasing general protein synthesis through phosphorylation of the initiation factor eukaryotic initiation factor 2 (eIF2*α*) which paradoxically increases selective translation of activating transcription factor 4 (ATF4) mRNA. The ATF4 protein is a member of the bZIP family of transcription factors that activates the expression of several UPR target genes involved in antioxidant responses, apoptosis, and autophagy. In ER stressed cells, ATF6 is cleaved at the Golgi apparatus, and the released cytosolic domain translocates to the nucleus where it increases the expression of ER chaperones, ERAD-related genes, and proteins involved in organelle biogenesis. However, when ER stress is prolonged or too severe, these adaptive mechanisms fail to restore protein-folding homeostasis, thus shifting adaptive programs toward the induction of apoptotic signaling to eliminate irreversibly damaged cells [107].

Unresolved and prolonged ER stress leads to perturbed Ca^2+^ homeostasis, increased protein accumulation, loss of ER function, and activation of apoptotic cascades [106]. Under these conditions, the level of the UPR-induced cell death mediator C/EBP-homologous protein (CHOP) increases [108] and activates the transcription of GADD34, which interacts with protein phosphatase I to catalyze eIF2*α* dephosphorylation [109, 110]. Dephosphorylated eIF2*α* in turn increases protein synthesis and oxidation leading to ER protein overload [111]. CHOP also represses the transcription of the antiapoptotic Bcl-2 protein [112]. Accordingly, deletion of CHOP gene partially protects both cells and animals from ER stress-mediated cell death [113]. The UPR is known to initiate other proapoptotic events as well, including c-Jun N-terminal kinase (JNK) phosphorylation, cleavage of ER-specific caspases such as caspase-12, and disruption of cellular Ca^2+^ homeostasis [114].

In the past few years, ER stress has been largely implicated in the pathogenesis of multiple human diseases, including neurodegenerative disorders [107, 115]. Several studies support that UPR activation upon ER stress is one of the main players in synaptic dysfunction and neuronal death occurring in AD [116–118]. In postmortem brain tissues from AD patients, a significant increase in the levels of ER stress markers, including phospho-PERK, phospho-eIF2*α*, and phospho-IRE1*α*, the transcription factor XBP1, the chaperone Grp78, and the downstream mediator of cell death CHOP has been reported, compared with age-matched controls, suggesting that the prolonged activation of the ER stress response is involved in the neurodegenerative process in AD [119–122]. Furthermore, recent studies revealed a connection between UPR activation and autophagic pathology in AD brain since the levels of microtuble-associated protein light chain 3 (LC3), an autophagosome marker, are increased in neurons displaying UPR activation [123]. Recent evidence obtained in an AD transgenic mice model, in which caspase-12, Grp78 and CHOP are strongly up-regulated, further implicates ER stress induction in the pathogenesis of AD [124]. Familial AD-linked presenilin-1 (PS-1) mutations downregulate the UPR and lead to ER stress vulnerability [125]. The mechanisms by which mutant PS-1 affects the ER stress response are attributed to the inhibited activation of ER stress transducers such as IRE1*α*, PERK, and ATF6. On the other hand, in sporadic AD, it was found that the aberrant splicing isoform (PS2V), generated by exon 5 skipping of the presenilin-2 (PS-2) gene transcript, downregulates the signaling pathway of the UPR [126].

Familial and sporadic AD are both associated with increased A*β* levels in brain parenchyma. Several evidences support that A*β* deposition and ER stress are interrelated events in AD. A global molecular profile of hippocampal and cortical gene expression revealed that ER stress-related genes are differentially regulated during the initial and intermediate stages of A*β* deposition [127]. ER stress was shown to enhance *γ*-secretase activity, as well as A*β* secretion [128]. On the other hand, it was proposed that A*β* is generated within the ER lumen as a result of deficits in axonal transport [129]. It was also found that in transgenic mice expressing APP(E693Δ) (APP(OSK)) intraneuronal A*β* oligomers accumulate in the ER in hippocampal neurons and cause cell death by inducing ER stress [130]. Additionally, the involvement of caspase-12 activation in A*β*-induced synaptic toxicity was recently demonstrated in cortical and hippocampal synaptosomes isolated from 3xTg-AD mice [131]. Several evidences demonstrate that A*β* is also able to trigger an ER stress response *in vitro* [132–134]. In primary cortical neurons, both fibrillar and oligomeric A*β* have been shown to upregulate Grp78 concomitantly with activation of the ER stress-mediated apoptotic cell death pathway [135, 136]. How A*β* causes ER stress is presently unclear. However, recent evidences obtained in cultured hippocampal neurons support that interaction of A*β* oligomers with NMDAR, in particular with the GluN2B subunits, occurs upstream of deregulation of ER Ca^2+^ homeostasis and upregulation of ER stress markers (Costa et al., unpublished data).

Perturbation of ER Ca^2+^ homeostasis, a trigger for the accumulation of unfolded or misfolded proteins and activation of the ER stress response, seems to play an important role in the onset or progression of neuronal dysfunction in AD [117, 137]. Significantly, a markedly decrease of calreticulin immunoreactivity (ER Ca^2+^ binding protein) was described in AD postmortem brain [138]. Recent studies in AD transgenic mice have shown that enhanced Ca^2+^ response is associated with increased levels of ryanodine receptors and altering synaptic transmission and plasticity mechanisms before the onset of histopathology and cognitive deficits [139, 140]. Moreover, mutant PS-1 interacts with the inositol-1,4,5-trisphosphate (IP_3_) receptor (IP_3_R)-associated Ca^2+^ release channel, resulting in Ca^2+^ signalling abnormalities [141, 142] that have been suggested to be an early pathogenic event in AD involved in presynaptic dysfunction [143]. Recently, it was discovered that PS-1 and PS-2 can form low-conductance channels, leading to passive ER Ca^2+^ leak [144]. These results provided potential explanation for abnormal Ca^2+^ signaling observed in familial AD cells with mutations in PSs. Several findings also implicate A*β* as a trigger of ER Ca^2+^ dyshomeostasis. APP overexpression was shown to potentiate CHOP induction and cell death in response to ER Ca^2+^ depletion [145]. Similarly, A*β* depletes ER Ca^2+^ through IP_3_R- and RyR-mediated Ca^2+^ release, thus increasing intracellular Ca^2+^ levels and compromising cell survival [136, 146]. In addition, A*β*-induced perturbation of intracellular Ca^2+^ homeostasis in neurons was shown to be correlated with an increase of the specific isoform of the ryanodine Ca^2+^ channel RyR3 expression and activity [147].

Recent evidences suggest that strategies able to ameliorate ER stress can prevent A*β* pathology. 4-Phenylbutyrate (PBA), acting through its chemical chaperone-like activity and via the transcriptional activation of a cluster of proteins required for the induction of synaptic plasticity and structural remodeling, was shown to mitigate ER stress. In the Tg2576 mouse model of AD, ER stress was accompanied by reversal of learning deficits, clearance of intraneuronal A*β* accumulation, and restoration of dendritic spine densities of hippocampal CA1 pyramidal neurons [148]. Additionally, the same authors demonstrated that chronic administration of PBA, starting before the onset of disease symptoms, prevents age-related memory deficits in Tg2576 mice, associated to a decrease in A*β* pathology and inflammation [148]. Wiley and colleagues [149] also demonstrated that PBA ameliorates the cognitive and pathological features of AD in the APPswePS1delta9 AD transgenic mice. In APP-overexpressing cells, PBA blocked the repressive effects of the ER stressors tunicamycin and thapsigargin upon APP proteolysis, UPR activation, and apoptosis [150]. Furthermore, silencing CHOP gene expression was shown to protect against AD-like pathology triggered by 27-hydroxycholesterol in rabbit hippocampus [151]. Recently, it was demonstrated that activation of the PERK-eIF2*α* UPR pathway prevents A*β*-induced neuronal ER stress [152]. Furthermore, the active form of the transcription factor XBP1 was shown to be neuroprotective in flies expressing A*β* and mammalian cultured neurons treated with A*β* oligomers, which was mediated by the downregulation of RyR3, preventing the accumulation of free Ca^2+^ in the cytosol [153]. In addition, dantrolene and xestospongin C, pharmacological inhibitors of ER Ca^2+^ release, were shown to prevent A*β*-induced apoptotic cell death [154, 155].

#### 4.1.2. ER-Mitochondria Crosstalk in AD

ER stress-induced apoptotic cell death involves a mitochondrial component [156, 157]. ER directly communicates with mitochondria through close contacts referred as mitochondria-associated membranes (MAMs) that promote Ca^2+^ transfer from ER to mitochondria thus maintaining mitochondrial metabolism and cell survival [158–160]. The molecular bridges that regulate the contacts between ER and mitochondria include the IP_3_R on the ER and the VDAC, which are physically coupled through the cytosolic chaperone glucose-regulated protein 75 kDa (Grp75) [161]. In addition, the dynamin-related GTPase mitofusin 2 (Mfn2) on the ER forms homoheterodimers with Mfn1 or Mfn2 on mitochondria to keep the tight contacts between the two organelles. Moreover, PACS-2 (mainly localized at the ER) and dynamin-related GTPase protein 1(Drp1) indirectly control the distance between the two organelles through regulation of mitochondrial morphology and distribution [162]. The chaperone Sigma-1 receptor (Sig-1R) is able to sense Ca^2+^ concentrations in the ER and controls the amount of Ca^2+^ released through the IP_3_R that can be transmitted to mitochondria [163].

Disruption of contact sites and impairment of Ca^2+^ coupling between ER and mitochondria have profound consequences for cellular function and in extreme cases lead to apoptosis. In fact, decreasing the space between both organelles promotes mitochondrial Ca^2+^ overload that can lead to the opening of the PTP, dissipation of the mitochondrial membrane potential and activation of apoptotic cell death [164], and, on the other hand, an increase in the distance between the two compartments inhibits Ca^2+^ transmission, compromising Ca^2+^-dependent regulation of mitochondrial metabolism and consequently cell viability [165]. Accordingly, during the adaptive phase of ER stress, an early increase in cellular bioenergetics and mitochondrial metabolism occurs [166] but during the cell death response, ER stress exerts profound deleterious effects on mitochondrial function [167] and activates an apoptotic pathway which depends crucially upon Ca^2+^ transfer from the ER to the mitochondria [135, 168]. The MAM is responsible for this transfer since its disruption, achieved by siRNA knockdown of PACS-2, results in the inhibition of ER Ca^2+^ release and apoptosis onset [162]. Furthermore, apoptotic stimuli known to act through Ca^2+^ release from the ER induce a prolonged increase in the mitochondrial Ca^2+^ concentration [154, 155, 169, 170].

Several members of the Bcl-2 family, such as Bcl-2 itself, Bax and Bak, naturally localize to both mitochondria and the ER and modulate Ca^2+^ content in both organelles, controlling the amount of ER-releasable Ca^2+^ that can reach mitochondria triggering apoptotic cell death [154, 171–176]. Transmission of a Ca^2+^ signal from ER to mitochondria was demonstrated to be associated with IP_3_-induced opening of PTP and, in turn, cytochrome c release [177]. Similarly, phosphorylation of IP_3_R by Akt reduces cellular sensitivity to apoptotic stimuli through a mechanism that involves diminished Ca^2+^ flux from the ER to the mitochondria [178]. Cytochrome c released from mitochondria can also bind to ER IP_3_R and promotes Ca^2+^ release through this channel [179]. Released ER Ca^2+^ triggers the extrusion of a large amount of cytochrome c from all the mitochondria in the cell, amplifying the death signal [180, 181]. It has been reported that mobilization of Drp1 to mitochondria, under ER Ca^2+^ release conditions, can trigger mitochondrial cristae remodelling, facilitating cytochrome c release and subsequent apoptosis [182, 183]. However, recruitment of Drp1 to mitochondria upon sustained Ca^2+^ release from the ER was described to protect from apoptosis by fragmenting the mitochondrial network and blocking Ca^2+^ transmission [184].

Despite the evidence that demonstrates the involvement of mitochondrial and ER dysfunction in AD pathogenesis [55], the role of ER-mitochondria crosstalk in this neurodegenerative disorder has not been clarified so far. It was recently shown that PS-1 and PS-2 are highly enriched in a subcompartment of the ER that is related with MAM [185]. In SH-SY5Y cells and primary neuronal cultures, overexpression of PS-2, and more drastically its familial AD mutants, was demonstrated to increase the physical interaction between ER and mitochondria thus facilitating mitochondrial Ca^2+^ uptake [186]. Moreover, the association of hyperphosphorylated tau with ER membranes was detected in AD brains and also in the brain of asymptomatic mice that overexpress mutant tau [187]. Interestingly, these mice exhibited more contacts between ER membranes and mitochondria, suggesting that accumulation of tau at the surface of ER membranes might contribute to tau-induced neurodegeneration through impairment of mitochondrial function [187]. Recent studies performed in mtDNA-depleted *ρ*0 cells challenged with toxic A*β* described the activation of an ER stress-induced apoptotic cell death pathway that requires the presence of a functional mitochondrial [188]. In A*β*-treated cortical neurons, it was previously demonstrated that Ca^2+^ released from ER, through IP_3_R and RyR channels [146], is implicated in the depolarization of the mitochondrial membrane, release of cytochrome c upon translocation of Bax to mitochondria and activation of caspase-9 [135, 136], thus implicating the ER/mitochondria crosstalk in neurodegeneration occurring upon A*β* exposure. This communication was also corroborated by the evidence obtained with cybrids, which recapitulate the mitochondrial defect (inhibition of complex IV of the electron transport chain) observed in AD [9, 189]. In these cells, markers of ER stress-induced apoptotic cell death were shown to be increased by A*β* treatment in comparison with controls suggesting that A*β*-induced ER stress is enhanced under mitochondrial dysfunction conditions [190].

### 4.2. ER-Driven ROS Production

Numerous evidences clearly implicate oxidative stress in AD pathogenesis. In this respect, the first thing that comes to our mind is mitochondrial-driven ROS generation. However, could ER be another important source of ROS in AD? Mainly during protein synthesis, 25% of cellular ROS are produced in the ER as a consequence of the activity of oxidoreductases, a family of proteins that catalyze protein folding reactions [191–193]. After the entry of nascent proteins in the ER, disulfide bond formation must occur to ensure their correct maturation and function. This reaction is catalyzed by the protein disulfide isomerase (PDI) that accepts electrons from thiol residues in the polypeptide chain substrate leading to its oxidation [194, 195]. To continue its activity, PDI must be reoxidized, a process that is guaranteed by oxidoreductin 1 (ERO1) [196]. In order to recycle itself, ERO1 transfers electrons to molecular oxygen, leading to the production of ROS. In AD patients, no substantial alterations were observed in PDI levels when compared to controls [197]; however this may not imply about its net activity. In fact, it was reported that the activity of PDI may be inhibited by ^•^NO, since increased levels of S-nitrosylated PDI were found in the brain of sporadic AD patients [198]. As a consequence, polyubiquitinated proteins accumulate, which may thus activate the UPR [198].

The ROS formation due to ERO1 activity is not exclusively linked to protein folding. ERO1 is retained in the ER through its interaction with PDI and the ERp44 [199, 200]. Beside this interaction, ERp44 also binds to the IP_3_R leading to its inhibition, a process that is dependent on pH, Ca^2+^ concentration and redox state [201]. In this way, ERp44 works as a sensor of the environment in the ER lumen. When this ERp44-IP_3_R connection is disrupted, ER Ca^2+^ is released through this channel into the cytosol. This process may rely on the presence of ERO1, since prolonged ERO1 activation is expected to originate a hyperoxidizing environment in the ER lumen [202], which may lead to the formation of disulfide bonds in the IP_3_R [201], disrupting the repressive interaction between ERp44 and IP_3_R [203]. Interestingly, ERO1*α*, one of the two ERO1 proteins expressed in human, was described to be localized on MAM [204], which is highly enriched in IP_3_R [205], suggesting that human ERO1*α* regulates IP_3_R-Ca^2+^ signaling on the MAM [204]. The Ca^2+^ released from ER can then enter directly into mitochondria, through the OMM VDAC or the IMM Ca^2+^ uniporter (MCU), leading to the increase in mitochondrial Ca^2+^ content [206], ROS production, and the opening of the PTP [207, 208]. This sequence of events is expected to occur in AD and can be hypothesized to underlie the increase in cellular ROS triggered upon ER Ca^2+^ release observed in A*β*-treated cortical neurons [135] (Figure 1).

Several ER functions, such as chaperone-mediated protein folding and refolding and the maintenance of Ca^2+^ gradients, are ATP-dependent processes. During the UPR, ER chaperones like Grp78 are upregulated, and consequently higher levels of ATP must be delivered to the ER, requiring an increase in ATP production by the mitochondrial respiratory chain, with the consequent enhancement of ROS production [209]. Similarly, the ER Ca^2+^ leak that occurs under prolonged stress conditions could obligate the sarco(endo)plasmic reticulum Ca^2+^-ATPase (SERCA) to increase the rate of entry of Ca^2+^ to the ER lumen, causing ATP depletion and subsequent increase of ROS production within the mitochondria.

Another consequence of UPR activation, in an attempt to recover from protein unfolding or misfolding, is the depletion of the antioxidant GSH. The function of GSH in the ER needs to be fully elucidated; however it has been suggested that GSH acts as a reductant [210], either by maintaining ER oxidoreductases in a reduced state or by directly reducing nonnative disulphide bonds in substrate folding proteins [211]. This may explain why the ER lumen contains a relatively high concentration of oxidized glutathione (GSSG), driving the GSH: GSSG ratio to approximately 3 : 1 [192, 212]. During UPR, the overload of unfolded proteins enhances ERO1 activity, leading to an increase in oxidized PDI levels, which requires higher levels of GSH. The subsequent conversion to GSSG leads to a depletion of the GSH pool. Another hypothesis for this decrease is that the stimulation of ERO1 activity increases the generation of ROS that reacts with GSH, decreasing its levels, which further increases ROS levels (Figure 1). In AD, contradictory results concerning GSH levels have emerged. Adams and colleagues [213] have suggested that GSH levels increase in the AD brain as a compensatory mechanism following damage in specific brain regions. In an opposite manner, Aksenov and coworkers [214] have reported that GSH metabolism is compromised in affected brain regions of AD patients. Moreover, GSH levels were described to be decreased in red blood cells from male AD patients and in experimental models of AD [215, 216]. It has been previously shown that GSH levels decrease in cortical neurons treated with A*β*, and this decrease was correlated with the release of Ca^2+^ from the ER [168]. This datum is further supported by previous results showing that depletion of GSH occurs in neurons treated with A*β* fibrils [217]. Therefore, A*β*-driven GSH depletion might contribute to the impairment of quality control mechanisms operating at the ER, leading to the accumulation of unfolded/misfolded proteins.

GSH is not the only antioxidant defense that may be reduced in AD as a consequence of ER stress and ROS formation. When the UPR is induced, the ER senses the increase in ROS and increases antioxidant defenses, namely, through the PERK signaling pathway that coordinates the convergence of ER and oxidative stress. One of these antioxidant responses involves the phosphorylation of Nrf2 by PERK, followed by its dissociation from the microtubule-associated protein Keap1 (Kelch-like Ech-associated protein 1), which allows the dislocation of Nrf2 from the cytosol to the nucleus [209, 218]. Once in the nucleus, Nrf2 binds to the antioxidant response element (ARE) to activate the transcription of several phase II detoxification enzymes and antioxidant enzymes [219]. Nrf2 activation also contributes to the maintenance of GSH levels, which in turn buffers the accumulation of ROS during the UPR [220]. Several studies allow us to speculate that the increase in ROS observed in AD may be linked, at least in part, to a deregulation of Nrf2 activity (Figure 1). Indeed, not only Nrf2 was described to be predominantly cytoplasmatic in hippocampal neurons from AD patients, resulting in decreased nuclear levels [221], but also Nrf2-ARE pathway was shown to be attenuated in APP/PS1 transgenic mouse brain at the time of A*β* deposition [222]. The potential protective role of Nrf2 in AD is further supported by the demonstration of a significant reduction in spatial learning deficits of aged APP/PS1 mice, observed when Nrf2 is overexpressed in this AD model [222].

When ER stress is prolonged, UPR signaling pathways ultimately lead to apoptosis. CHOP is one of the mediators of ER stress-mediated apoptotic cell death. Li and colleagues [223] have demonstrated that CHOP induces ERO1*α* upregulation, which causes the activation of the ER IP_3_R. The Ca^2+^ released from ER can enter the mitochondria, promoting ROS generation as described above, but can also activate the enzyme calcium/calmodulin-dependent protein kinase II (CaMKII), which triggers mitochondrial-mediated apoptosis [224] (Figure 1). CaMKII can further induce NOX that activates a protein kinase R (PKR-) activating protein, leading to sustained PKR-mediated CHOP expression, amplifying the pathway induced by this ER stress-related transcription factor [225]. In AD patients, during the initial stages of the disease, the expression of all 3 isoforms of NOX was shown to be significantly increased [226], activating NOX-associated pathways and contributing to AD progression [45]. The connection between CHOP upregulation and NOX signaling in AD remains to be further clarified but it seems to be a good target for future therapeutic perspectives. Another positive feedback is played by ROS itself that can sensitize both Ca^2+^-release channels and SERCA at the ER membrane [227–229]. ROS or RNS can oxidize critical thiols in the RyR, causing Ca^2+^ release [230]. On the other hand, oxidation of SERCA inhibits their ability to transport Ca^2+^ to the ER lumen, increasing cytosolic Ca^2+^ concentration [228].

From the data exposed above it is possible to conclude that the ER could be, by its nature, an important source of ROS in AD, which impacts on cell survival upon perturbation of normal ER function. Due to the close communication between ER and mitochondria, ER stress occurring in AD brain can be expanded to the mitochondria releasing its malicious oxidative power that can further trigger apoptotic cell death pathways. Therefore, targeting these cellular sources of ROS may bring strong therapeutical outcomes for this neurodegenerative disease.

## 5. Oxidative Stress Markers in Biological Fluids from AD Patients

With the move towards development of disease-modifying treatments, there is a need for more accurate diagnosis of AD in its early stages. Therefore, much attention has been paid to the identification and validation of biological markers of the disease. Markers that specifically reflect the onset of pathology may have a profound impact both on early diagnosis and on detection of treatment effects in the near future. Established CSF biomarkers exist for early AD: total and hyperphosphorylated tau (tau and p-tau) that reflect AD-type axonal degeneration and the 42 amino acid isoform of amyloid *β* (A*β*1-42) that reflects senile plaque pathology [231]. These biomarkers have recently been incorporated in the new proposed revised criteria for AD [232, 233]. However, these classical markers do not capture all the pathological changes that take place in the brain of AD patients, and its clinical application is limited by the invasive nature of its collection. Impaired bioenergetics, increased production of ROS, and oxidative injury are, as seen above, important features of AD pathology that occur early in the course of the disease. These findings have spurred the development of assays for markers that reflect these processes both in tissue, CSF and peripheral fluids.

The methodology mostly used to assess oxidative damage is through the detection of products of free radical attack against biomolecules (lipids, proteins, and nucleic acids). Additionally, several compounds of the antioxidant defense system can be measured and used as complementary information regarding the oxidant/antioxidant balance of the organism.

Results on lipid peroxidation in plasma and peripheral blood cells have been inconsistent, with several authors demonstrating increased levels of free MDA or TBARS in serum/plasma [234–239] or in erythrocytes [240, 241] of AD patients and MCI subjects, whereas others did not confirm these findings [242–244]. Interestingly, a few studies have shown that the highest TBARS levels were found in APOE-*ε*4 carriers [13, 241], suggesting that APOE genotype affects the extent of the oxidative stress-induced damage.

Free HNE has also been assessed in ventricular CSF from patients with AD, and significantly elevated levels were found in comparison to age-matched controls, while no differences were detected in the levels of HNE-protein adducts [245]. Similar to what has been reported for MDA and TBARS, the results of the determination of HNE in peripheral fluids of AD and MCI subjects have been somewhat inconclusive. Some authors have demonstrated elevated plasma levels of HNE in AD patients, compared to controls [243, 246], while others did not observe any differences [247]. An interesting study [248] reported increased levels of MDA and HNE in peripheral cells (skin fibroblasts and lymphoblasts) derived from familial AD patients, carrying APP and PS-1 mutations, while no differences in these lipid peroxidation markers were found between sporadic AD cases and controls.

Increased levels of F_2_-IsoPs were also found both in postmortem ventricular CSF from AD patients [20, 249] and in lumbar CSF collected *in vivo* [250, 251], correlating with clinical severity and other biomarkers of the disease, like CSF A*β*1-42 and tau [252, 253]. Several studies on MCI subjects also found increased levels of CSF F_2_-IsoPs [251, 254, 255], including longitudinal studies, that have shown that CSF F_2_-IsoPs levels rise after 12-month followup [254] and that the rate of increase is higher in MCI subjects that progress to AD, compared to healthy controls and stable MCI [256]. In fact, longitudinal evaluation of CSF F_2_-IsoPs seems to be useful in predicting future cognitive deterioration both in cognitive normal and MCI subjects and in increasing the diagnostic accuracy of prodromal AD [255, 257]. One study in particular [258] suggests that the determination of CSF isoprostanes could be useful in monitoring the effectiveness of experimental antioxidant treatments. The quantification of isoprostanes in peripheral fluids of AD patients and MCI subjects has however yielded conflicting results. Some studies have found elevated levels of F_2_-IsoPs in the urine [252, 259] and plasma of AD patients [252] and MCI subjects [251], but further studies did not confirm these results [260, 261].

Overall, it seems that data regarding oxidative damage to lipids in the central nervous system is fairly consistent in showing increased markers of lipid peroxidation in early stages of AD. However, when moving to peripheral fluids, results are rather conflicting. Methodological differences could in part explain these contrary results. Furthermore, multiple physiological and pathological conditions can influence the levels of lipid oxidative damage in peripheral fluids, such as diet, physical activity, smoking habits, and comorbidities like diabetes, cardiovascular disease, and cancer that are known to increase oxidative damage. Therefore, when analysing the levels of lipid peroxidation markers in peripheral fluids in AD patients and MCI subjects, it is extremely important to control for potential confounders.

Protein carbonyls are usually detected with 2, 4-dinitrophenylhydrazine (DNPH) by a simple spectrophotometric assay. Carbonyl content has also been studied in plasma, with some studies failing to show an increase in this protein oxidation marker in AD patients [243, 262] and MCI subjects [241]. Recent studies, however, have demonstrated increased plasma concentrations of protein carbonyls in AD patients and MCI subjects, compared to controls [263], and also in peripheral lymphocytes isolated from AD patients [246].

Protein nitration, detected by nitrotyrosine immunoreactivity, has been studied not only in the brain but also in CSF. By employing sensitive HPLC methods, five-to eightfold increases in the levels of 3-NT have been found in the ventricular and lumbar CSF of AD patients when compared with cognitively normal controls [264, 265]. These results, however, were not confirmed by a different study using a gas chromatography coupled with mass spectroscopy approach [266], where the majority of AD patients had 3-NT CSF levels similar to the controls. The discrepancies between these studies are probably due to the different sample preparation and analysis methods and to the possible *in vitro* formation of 3-NT in the CSF samples. Similarly to what has been shown for protein carbonyls, increased levels of 3-NT have also been reported in plasma and lymphocytes of AD patients compared to controls [124].

 DNA injury, assessed through increased levels of 8-OhdG, has also been shown in intact DNA extracted from ventricular CSF [267, 268] or in lumbar CSF of AD patients [269]. Studies using DNA extracted from peripheral tissue have also demonstrated increased levels of DNA oxidation, thus suggesting the systemic nature of oxidative damage in AD. Increased levels of 8-OHdG and oxidized purines and pyrimidines in the peripheral lymphocytes and leukocytes of AD and MCI patients have been demonstrated [235, 270, 271] and also an increased urinary excretion of oxidized nucleosides in AD patients [272]. The potential of DNA oxidation levels as a biomarker for AD has been questioned, however, due to the overlap between AD and controls and to its lack of specificity, as increased DNA oxidation seems to be present in other neurodegenerative conditions, such as amyotrophic lateral sclerosis and Parkinson's disease [272]. Besides DNA, oxidation of RNA can also be used as a marker of oxidative stress, through the determination of 8-OHG levels. Interestingly, those were found to be fivefold increased in the CSF of AD patients compared to controls, being unaltered in the serum [273].

Contradictory results have been reported regarding the peripheral activity of cellular antioxidant enzymes in AD patients. While some studies have not found any differences in the activity of these enzymes in red blood cells of AD or MCI subjects as compared to controls [234, 241, 242], others have reported an increased activity of glutathione peroxidase, catalase, and superoxide dismutase [236, 239, 240], but decreased activity of the latter enzymes has also been found [238, 274, 275]. Regarding nonenzymatic antioxidants, including glutathione, uric acid, carotene, lycophene, vitamins A, C, and E, work from several groups has demonstrated decreased plasmatic levels in AD patients [234, 269, 276] and MCI subjects [263, 275, 276], with some authors suggesting that progression to AD might be related to depletion of antioxidant defenses [277]. One of the most investigated nonenzymatic antioxidants is probably vitamin E, the most powerful chain-breaking antioxidant [278], with reduced levels reported not only in plasma [234, 241, 275, 276] but also in CSF [279] and brain parenchyma of AD patients [280]. Antioxidant intervention in animal models of AD showed a significant reduction in oxidative stress, A*β* deposition, and also behavioral improvements [281, 282]. However, in AD clinical trials, antioxidants have shown only a marginal positive effect on disease progression [283, 284], and subsequent MCI trials with antioxidants indicate that vitamin E ingestion has no benefit on the risk of progression to AD [285, 286]. The lack of success of these trials [287–289] likely arises from a combination of factors, including using the wrong dose in an unbalanced monotherapy, not monitoring the drug levels and surrogate markers for the *in vivo* therapeutic effect of the drug of interest and starting the therapy very late in the disease stage. The failure of simple antioxidants to reverse ROS damage has prompted the need of other mitochondrial-targeted therapies, such as acetyl-L-carnitine-carnitine (a compound that acts as an intracellular carrier of acetyl groups across the inner mitochondrial membrane), MitoVitE (a compound that results from the conjugation of vitamin E with the lipophilic triphenylphosphonium cation—TPP+—making the antioxidant selectively accumulate inside the mitochondria), Szeto-Schiller peptides (small cell permeable antioxidants that target mitochondria in a potential-independent manner), or Dimebon (the Russian antihistamine laterpirdine), as reviewed elsewhere [290, 291].

The failure of antioxidant therapy to attenuate disease progression [285, 286] might also be explained by the fact that oxidative stress could be a necessary but insufficient factor for the development of disease, that is dependent upon additional factor(s) for the onset of underlying pathogenesis. Nevertheless, early intervention to prevent chronic oxidative stress, and thereby ameliorate one of the factors for the development of the disease, should influence and reduce the risk of ever developing the disease. Indeed, the role of oxidative stress in the pathogenesis of AD has moved from an epiphenomenon to one of the earliest events in disease pathogenesis, occurring prior to the onset of symptoms and associated with the brain regions typically affected in the disease [14, 28, 38–40, 43, 251]. The hypothesis, based on *in vitro* cell culture experiments, that A*β* causes oxidative stress [1] has been challenged by *in vivo* studies where oxidative stress chronologically precedes A*β* deposition. In fact, A*β* accumulation is associated with reduced levels of oxidative stress [38–40]. Therefore, the identification of valuable reliable peripheral markers of oxidative damage would be of utmost importance for researchers and clinicians. Currently there isn't no single biomarker of oxidative stress. The standardization of assessment methods and the consideration of potential confounders are critical to reduce the inconsistencies that have been reported between studies. Moreover, many of these studies have been done by comparing AD patients and/or MCI subjects with healthy controls and not with other neurodegenerative diseases, so specificity is still an issue. Oxidative stress has been found increasingly implicated in a number of neurodegenerative disorders including AD, Parkinson's disease (PD), and amyotrophic lateral sclerosis (ALS) [292]. However, even if a process is not specific to AD pathogenesis, such as oxidative damage, its biomarkers may be useful in the context of clinical and imaging studies to monitor disease progression and optimize therapy. Increased sensitivity and specificity can probably be achieved by using a panel of different biochemical indices that target different pathological processes and can provide a more accurate picture of the oxidative balance of the organism.

## 6. Concluding Remarks

Alzheimer's disease (AD) is the most common age-related dementia. It is a slowly progressive and chronic neurodegenerative disorder, in which cognitive impairment is related to synapses degeneration and neuronal death occurring in the limbic system and specific regions of the cerebral cortex. The accumulation of A*β* in senile plaques and the intraneuronal aggregates of hyperphosphorylated tau protein are recognized hallmarks of the disease whose cause still remains unknown.

Several lines of evidence show that mitochondria dysfunction, Ca^2+^ deregulation, and oxidative stress are prominent factors in AD cellular pathology. Mitochondria, where free oxygen radicals are generated as by-products from the electron transport chain and from enzymes of the tricarboxylic acid cycle, are main sources and simultaneously main targets of ROS.

Toxic A*β* oligomers may induce Ca^2+^ influx into neurons, rendering neurons vulnerable to excitotoxicity, through the activation of glutamate NMDAR, and apoptosis. Glutamate excitotoxicity and/or oxidative stress have been shown to alter mitochondrial fission/fusion and an imbalance in mitochondria dynamics in turn leads to NMDAR upregulation and oxidative stress. In addition, A*β* accumulates in mitochondria and thereby impairs the activity of mitochondria respiratory chain and reduces ATP synthesis and the mitochondria Ca^2+^ buffering capacity, causing elevated cytoplasmic Ca^2+^ levels and oxidative stress.

A*β* was also shown to promote ER stress and excessive release of Ca^2+^ from ER which may underlie mitochondrial Ca^2+^ dyshomeostasis and ROS generation, thereby disturbing organelle functioning and, ultimately, damaging neurons.

Mitochondria and the ER are closely linked morphologically and functionally, and considerable crosstalk of cell death proteins, promoted by ROS and high Ca^2+^ levels, occurs between these two organelles. The Ca^2+^ transport systems of the ER are also sensitive to oxidative stress being directly exposed to ER/mitochondria-generated ROS. The resulting abnormal cellular Ca^2+^ load can trigger cell death by activating proteases, reinforcing signals leading to caspase activation, such as cytochrome c release from mitochondria, or by triggering other catabolic processes mediated by lipases and nucleases.

A*β*-associated Ca^2+^ deregulation, impaired bioenergetics, increased production of ROS, and oxidative injury to lipids, proteins, and nucleic acids, associated to impairment of antioxidant defences, are important features of AD cellular pathology that occur early in the course of the disease. It can be hypothesized that the progression to AD may be related to the incapacity of the antioxidant system to counterbalance the oxidative injury, leading to disruption of cell redox signaling. In this context, development of reliable oxidative stress biomarkers and new antioxidant strategies should be proposed as primary prevention measures, even before significant plaque deposition or cognitive decline.

## Abbreviations

ATF6: Activating transcription factor 6
CaMKII: Calcium/calmodulin-dependent protein kinase II
CHOP: C/EBP-homologous protein
ER: Endoplasmic reticulum
GSH: Glutathione
IP_3_R: Inositol-1,4,5-trisphosphate receptor
IRE1*α*: Inositol-requiring enzyme 1 *α*
MCU: Mitochondrial Ca^2+^ uniporter
NOX: NADPH oxidase
NMDARs: *N*-methyl-D-aspartate receptors
Nrf2: Nuclear factor (erythroid-derived 2)-like 2
PDI: Protein disulphide isomerase
PERK: Protein kinase R-like endoplasmic reticulum kinase
ROS: Reactive oxygen species
UPR: Unfolded protein response
VDAC: Voltage-dependent anion channel.
